# Titanium prostheses versus stapes columella type 3 tympanoplasty: a comparative prospective study

**DOI:** 10.1016/j.bjorl.2020.07.014

**Published:** 2020-09-14

**Authors:** Fayez Bahmad, Andréa Gonçalves Perdigão

**Affiliations:** a Hospital Universitário de Brasília, Brasília, DF, Brazil; b Universidade de Brasília (UnB), Faculdade de Ciências de Saúde, Programa de Pós-Graduação em Ciências da Saúde, Brasília, DF, Brazil; c Universidade de Brasília (UnB), Faculdade de Medicina, Brasília, DF, Brazil

**Keywords:** Tympanoplasty, Prostheses, Stapes, Otitis media

## Abstract

**Introduction:**

Tympanoplasty is a surgical procedure designed to reconstruct the mechanisms of sound transmission in the middle ear.

**Objective:**

Analyze, from an audiological point of view, patients with chronic otitis media undergoing type 3 tympanoplasty major columella with total ossicular replacement titanium prosthesis or with cartilage graft stapes columella.

**Methods:**

This is a prospective analytical study, carried out at the otorhinolaryngology outpatient clinic in a tertiary care hospital, through the evaluation of 26 patients with chronic otitis media who underwent tympanoplasty using different materials for auditory rehabilitation such as titanium prostheses or cartilage autografts.

**Results:**

There was no statistically significant association between the group factors (cartilage or titanium reconstruction) and preoperative variables. There was no statistically significant association between the postoperative characteristics of the patients and the type of reconstruction. Neither subjective improvement (hearing improvement) nor residual perforation were associated with a type of material. The via factor was the only one that showed a statistically significant difference once air-conduction pathway improved more than bone-conduction pathway, decreasing the air-bone gap.

**Conclusion:**

There was no statistical difference between the two groups in relation to the audiometric improvement. There was hearing improvement in both groups. More studies must be done with a longer follow-up to better evaluate the outcome.

## Introduction

Tympanoplasty is a surgical procedure developed to reconstruct the mechanisms of sound transmission in the middle ear. The new era of tympanoplasty began in 1950 with the pioneering work of Wüllstein^1^ and Zollner.^2^ Many other otologists then contributed to the development and refinement of tympanoplasty techniques. Wüllstein classified the operation techniques from Type I to Type V and we adopted a modified version of his classification proposed by Merchant in 2003 (Table 1).^1^, ^2^, ^3^, ^4^, ^5^

**Table 1:** Wüllstein modified by merchant classification.

Type of tympanoplasty	Middle ear pathology	Graft
**Tympanoplasty + Mastoidectomy**		
Canal wall-up:		
Type I	TM perforation; intact and mobile ossicles	Temporalis fascia graft; perichondrium; cartilage
Type II	Small ossicular discontinuity (along incus’ long process and stapes)	Cartilage; mastoid bone
Type III: minor columella	Diseased malleus and incus; intact and mobile stapes, intact posterior canal wall	Autologous ossicles, autologous cortical skull bone, hydroxyapatite or titanium PORP
Type III: major columella	Absent stapes superstructure, footplate mobile	Hydroxyapatite or titanium TORP between the footplate and the TM/manubrium
**Tympanoplasty + Mastoidectomy**		
Canal wall-down:		
Type III: stapes columella	Intact and mobile stapes	Thin cartilage disk + temporalis graft onto the stapes head
Type III: major columella	Absent stapes superstructure, mobile footplate, deep and narrow oval window niche	Hydroxyapatite or titanium TORP + thin cartilage disk + fascia graft
Type IV	Absent stapes superstructure, mobile footplate, shallow and wide oval window niche	Thin skin graft over the footplate, thick cartilage + temporalis fascia to shield the round window
Type V	Fixed footplate	Second stage total spadectomy with adipose graft and thick cartilage fascia to shield the round window

TM, Tympanic Membrane; TORP, Total Ossicular Replacement Prosthesis; PORP, Partial Ossicular Replacement Prosthesis.

In Chronic Otitis Media (COM) patients, there are three main goals to achieve in tympanomastoidectomy: 1) eradicate the disease to keep an aerated and dry ear 2) modify the structure to avoid recurrent disease and improve monitoring, and 3) restore the middle ear structure to reestablish the hearing function. Even though this article is focused on the third goal, we must emphasize that the technique employed was chosen considering the first two goals.^5^, ^6^, ^7^

In a healthy ear, the sound waves received from the air moves the tympanic membrane (TM). This acoustic energy flows through all the ossicular chain, concentrating the energy from a bigger surface, the TM, to a smaller one, the oval window. When this mechanism is impaired, we choose to perform a tympanoplasty, which aims to reconnect the TM and the ossicular chain, so it is mobile and able to reestablish the sound pressure at the oval window membrane. The improvement is more significant in frequencies between 250 and 1000 Hz, varying around 20 dB.^1^, ^2^, ^3^, ^4^, ^5^, ^6^, ^7^

This scientific article aims to analyze, from an audiological point of view, patients with COM divided into two groups: group 1 undergoing Type III tympanoplasty major columella with total ossicular replacement prosthesis (TORP) of titanium, and group 2 undergoing stapes columella reconstruction with an autologous cartilage graft. The pure-tone audiometry tests were performed 6 months before and after the surgery on both groups; the followup period was 6 months.^1^, ^2^, ^3^, ^4^, ^5^, ^6^, ^7^

## Methods

This is a prospective analytical study carried out with 26 patients with COM, treated at a tertiary reference center. Patients underwent a canal wall down (CWD) tympanoplasty and mastoidectomy using different auditory rehabilitation methods and materials, divided into two groups: group 1 submitted to Type 3 Tympanoplasty TORP with titanium prostheses (major columella) and Group 2 undergoing reconstruction with autologous cartilage (stapes columella).^6^, ^7^, ^8^

Inclusion criteria were patients with COM undergoing to type 3 tympanoplasty major columella or stapes columella that maintained tympanic cavity dried and aerated after careful examination of the researcher. The exclusion criteria were patients who abandoned outpatient followup within 6 months after surgery. All the patients included agreed to sign the written informed consent form (WICF), which reassures the voluntary nature of participation and clarifies all the procedures and its safety to the participant.

This project was approved by the Research Ethics Committee (REC) of the Health Sciences Faculty. As it was a research involving human beings, the ethical aspects disciplined by Resolution 466/12 of the National Health Council/Ministry of Health, assuring to the participants all information about research purpose, anonymity, free consent, and the freedom to give up the participation at any stage of the research. The Free and Informed Consent Form was available for the participants to sign, who had their rights guaranteed. Participants were aware that the pre and postoperative audiologic parameters would be analyzed, being thus free from risks during the research. The researcher is responsible for the confidentiality of patient's information.(This entire paragraph is redundant and simply restates comments made in the preceding paragraph. It should be deleted.)

The instruments of the research were questionnaires previously tested and validated, which were applied before and after patients were submitted to the tympanoplasty for auditory rehabilitation. All pure-tone audiometry tests were done at the otorhinolaryngology department of the reference center. Preoperative and postoperative audiometric data, intraoperative findings and the type of prosthesis used were recorded. The following frequencies were evaluated on pre and postoperative tests: 500, 1000, 2000 and 3000 Hz. The pure tone average (PTA) was calculated using the frequencies 500, 1000, 2000, 3000 Hz.

After collecting all the data through the questionnaire, it was processed using Excel software for Windows. Statistical analysis was performed with SPSS software (Statistic Package for the Social Sciences, Chicago, IL, USA) version 13 for Windows. The possible associations between the group and the pre- and postoperative variables were evaluated using the Chi-Square test.

The possible associations between the group and the pre- and postoperative variables were evaluated using the chi-square test. Correction of the level of statistical significance in all 2 × 2 contingency tables was done using Fisher's exact test.

Comparisons of mean age, pure tone audiometry test during the first session and mean difference in Speech Recognition Threshold (SRT) (the lowest sound level in which words and syllables may be identified) between groups were done with the *t*-test for independent measurements.

In order to eliminate a factor (session) in the mixed-design analysis, the before and after difference was calculated for each of the frequencies, pure tone average and SRT evaluated in pure tone audiometry. This approach also has the advantage of allowing direct comparisons between frequencies and conduction pathways and eliminating differences that may be present from the beginning of the study.

The analysis of the mean differences (before and after) was done with a split-plot ANOVA (mixed-design analysis of variation) model using the group factors (reconstruction with titanium or cartilage) as independent variables; via (air and bone) and frequency factors (500, 1000, 2000 and 3000 Hz) as repeated measures. In the analysis of the pure tone average the frequency factor was withdrawn.

The Greenhouse-Geisser method was used to correct the degrees of freedom (sphericity not assumed), but the original values ​​of degrees of freedom are presented. The multiple comparisons procedure used the Bonferroni method to correct the level of statistical significance.

We also tested whether the mean differences were statistically different from 0 using the *t*-test for a sample. The results are displayed as mean and standard error. The level of statistical significance was set at 5% (*p* <  0.05).

## Results

There was no statistically significant association between the patients’ preoperative characteristics and the reconstruction material used. Sex, operated side, previous surgery, cholesteatoma association, incus long process erosion and associated characteristic of COM did not influence inclusion in titanium group (titanium reconstruction) or cartilage group (cartilage reconstruction) (Table 2).

**Table 2:** Results of the association between patient’s preoperative characteristics and the Group factor (reconstruction material).

Variable	Reconstruction	Cartilage	Reconstruction	Titanium	X^2^	*p*-value
	n	%	n	%		
Sex						
Female	3	23.0	6	46.2	0.119	0.749
Male	10	77.0	7	53.8		
Operated side						
Right	7	53.8	6	46.2	0.073	>0.99
Left	6	46.2	7	53.8		
Previous surgery						
No	11	84.6	11	84.6	0.588	0.512
Yes	2	15.4	2	15.4		
Cholesteatoma						
No	7	53.8	6	46.2	0.029	>0.99
Yes	6	46.2	7	53.8		
Incus’ long process erosion						
No	3	23.0	0	0.0	1.867	0.287
Yes	10	77	13	100.0		
Diagnosis						
COM	0	0.0	0	0.0	7.667	0.105
COM + Cholesteatoma	6	46.15	7	53.84		
Simple COM	4	30.76	6	41.15		
Suppurative COM	3	23.07	0	0.0		
Trauma	0	0	0	0.0		

COM, Chronic Otitis Media; n, number.

**Figure 1:**
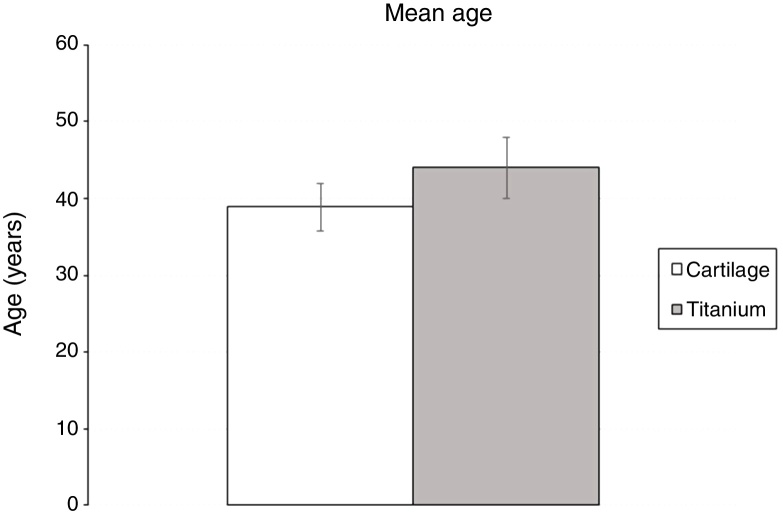
Mean of the patients’ age submitted to middle ear reconstruction using titanium or cartilage (Group 1 or Group 2). There is no statistically significant difference between the two groups based on age (38.9 ± 3.1 e 44.0 ± 4; *p* =  0.316).

There was also no difference between the mean age of patients submitted to reconstruction with cartilage or titanium (38.9 ± 3.1 e 44.0 ± 4; *p* =  0.316) (Fig. 1).

There was no statistically significant association between the postoperative characteristics of the patients and the group factor. Despitea different number of patients acheiving hearing improvement and residual perforation between the groups; the difference was not statistically significant (Table 3).

**Table 3:** Results of the association between the group factor and the postoperative characteristics of the sample.

Variable	N	%	X^2^	*p*-value
**Procedure**				
TM canal wall down	26	100		
TM CWD + Type 3 Tympanoplasty: stapes columella	13	50		
TM CWD + Type 3 Tympanoplasty: titanium TORP (Kurz)	13	50		
**Reconstruction**				
Concha cartilage/stapes	4	15.4		
Tragus cartilage/stapes	9	34.6		
Titanium TORP (Kurz)	13	50		
**Hearing Improvement**				
TM CWD + Type 3 Tympanoplasty: stapes columella				
Yes	10	77		
No	3	23		
			1.202	0.388
TM CWD + Type 3 Tympanoplasty: titanium TORP (Kurz)				
Yes	13	100		
No	0	0		
**Residual Perfuration**				
TM CWD + Type 3 Tympanoplasty: stapes columella				
Yes	2	15.4		
No	11	84.6		
			0.032	>0.99
TM CWD + Type 3 Tympanoplasty: titanium TORP (Kurz)				
Yes	1	7.7		
No	12	92.3		

TM, Tympanic Membrane; CWD, Canal Wall Down; TORP, Total Ossicular Replacement Prosthesis.

### Results of audiometric tests

The results of the preoperative pure-tone audiometry in each group showed no statistically significant association on most of the frequencies measured, on both conduction pathways. Only one slightly significant difference was found between the groups in the 1000 Hz frequency recorded by the bone-conduction pathway (Table 4).

**Table 4:** The mean of the patients’ preoperative pure-tone audiometry tests (air-conduction and bone-conduction threshold) on four different frequencies and the pure-tone average on both groups (Cartilage and Titanium). The *p*-value refers to the comparison of means between groups.

Variable	Cartilage		Titanium		*p*-value
	Mean	SE	Mean	SE	
Air conduction					
500 Hz	52.9	2.9	51.3	4.0	0.750
1000 Hz	53.7	3.4	49.0	5.1	0.434
2000 Hz	47.3	2.1	45.0	4.8	0.664
3000 Hz	49.2	2.6	44.0	4.8	0.304
Pure-tone average	50.8	2.4	47.3	4.4	0.502
Bone conduction					
500 Hz	14.0	2.1	9.0	1.6	0.102
1000Hz	15.6	2.4	8.3	1.8	0.020
2000 Hz	17.7	2.3	16.0	3.4	0.673
3000 Hz	21.3	3.2	15.3	3.4	0.230
Pure-tone average	17.2	2.3	12.2	2.3	0.156
SRT	51.9	2.9	50.3	5.6	0.802

SRT, Speech Recognition Threshold; SE, Standard Error.

**Figure 2:**
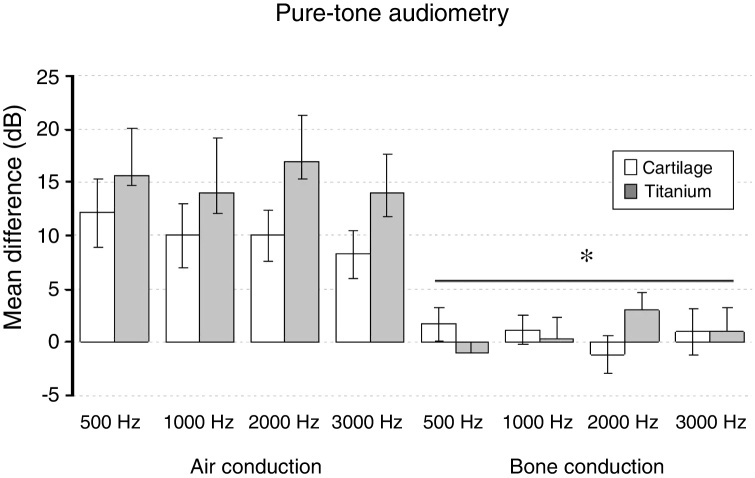
The mean of the difference before-after surgery and standard error of air and bone-conduction threshold for each frequency evaluated by pure-tone audiometry on both groups. * Bone pathway < airway (*p* <  0.001).

**Figure 3:**
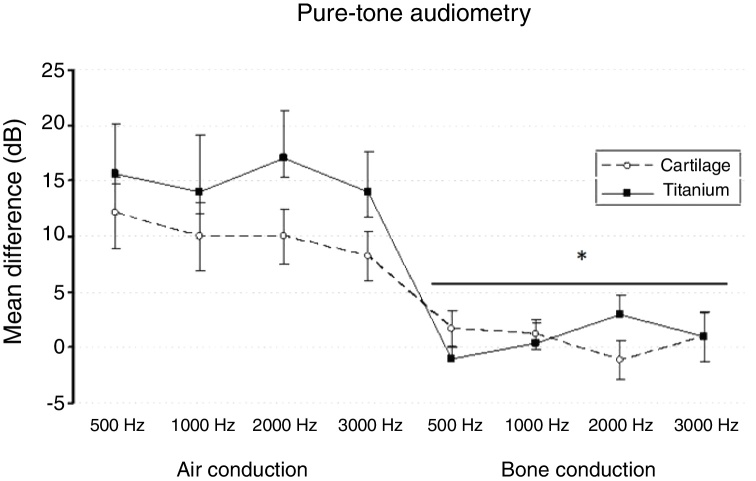
The mean of the difference before-after surgery and standard error of air and bone-conduction threshold for each frequency evaluated by pure-tone audiometry in both groups. * Bone pathway < airway (*p* <  0.001).

The split-plot ANOVA did not find a statistical significant effect of the group factor (titanium or cartilage) on the mean of the difference (before − after) of the pure-tone audiometry results (F_1,39_ = 0.979, *p* =  0.329). However, the mean of the difference (before and after) of the air conduction threshold is statistically significant in every frequency evaluated for both materials, which indicates that the null hypothesis – no hearing improvement − is not likely to occur. There was a statistically significant difference when analyzing the via factor (Air or bone conduction pathways) (F_1,39_ = 28.316, *p* <  0.001) (Table 5). The multiple comparisons procedure showed that the mean of the differences of the air conduction threshold were significantly higher than the ones recorded by the bone conduction threshold, regardless of group or frequency evaluated (*p* <  0.01). The frequency factor and the interactions Via × Group, Frequency × Group, Via × Frequency and Via × Frequency × Group had no significant effect on the mean difference (F < 1.665, *p* > 0.188 in all cases) (Figure 2, Figure 3).

**Table 5:** The mean of the differences (before − after) and the standard error of the air-conduction threshold and bone-conduction threshold for different frequencies on both groups (cartilage and titanium) and the pure-tone average. The *p*-value refers to the comparison against the 0 value (*t*-test of a sample).

Mean difference	Cartilage		*p*-valor	Titanium		*p*-value
	Mean	SE		Mean	SE	
Air conduction						
500 Hz	12.1	3.1	0.001	15.7	4.4	0.003
1000 Hz	10.0	3.1	0.003	14.0	5.2	0.017
2000 Hz	10.0	2.4	0.000	17.0	4.3	0.002
3000 Hz	8.3	2.2	0.001	14.0	3.6	0.002
Pure-tone average	10.1	2.4	<0.001	15.2	4.1	0.002
Bone conduction						
500 Hz	1.7	1.6	0.280	−1.0	1.0	0.334
1000 Hz	1.2	1.4	0.407	0.3	2.0	0.869
2000 Hz	−1.2	1.8	0.523	3.0	1.7	0.108
3000 Hz	1.0	2.2	0.661	1.0	2.2	0.663
Pure-tone average	0.7	1.4	0.635	0.8	1.4	0.006
SRT	11.2	3.1	0.001	16.2	5.0	0.554

SRT, Speech Recognition Threshold; SE, Standard Error.

**Figure 4:**
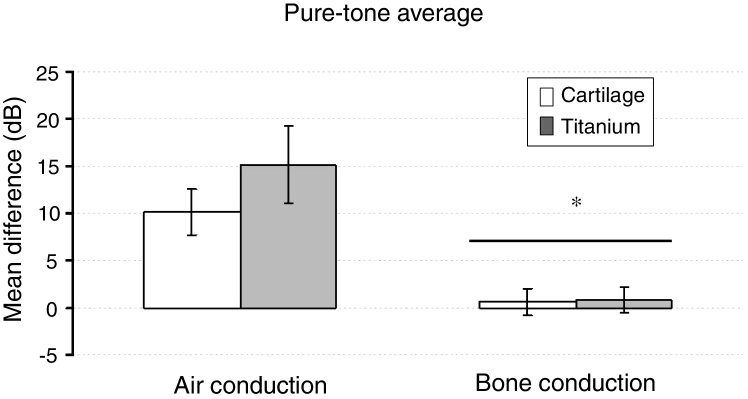
The mean of the difference before-after and standard error of pure-tone average evaluated based on air and bone-conduction threshold in both groups. * Bone pathway < airway (*p* <  0.001).

The pure tone average did not show a significant effect on the group factor (F_1,39_  = 0.979, *p* =  0.329). But, based on the pure-tone audiometry tests, the via factor had a significant effect on the mean difference (F_1,39_ = 28.316, *p* < 0,001). The preoperative air-bone gap (ABG) of 33.6 ± 4.7 lowered to 24.2 ± 8.5 on the cartilage group, and from 35.1 ± 6.7 to 20.7 ± 12.2 on the titanium group (*p* < 0.01). The air-conduction threshold had a significantly greater mean difference compared to the bone-conduction (*p* <  0.001). The Group × Via interaction had no significant effect (F_1,39_ = 1.210, *p* =  0.278) (Fig. 4).

**Figure 5:**
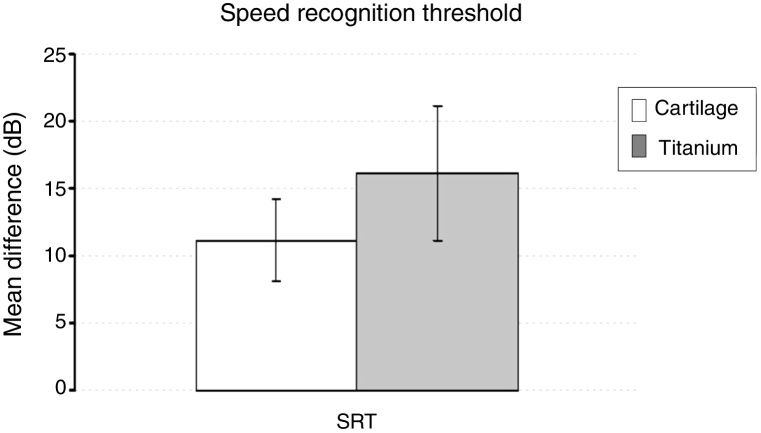
The mean of the difference before-after and standard error of Speech Recognition Threshold (SRT) assessed by pure-tone audiometry for each group. * Not statistically significant; *p* >  0.05.

There was no significant difference between the groups in the mean speech recognition threshold (SRT) difference (t = −0.911, *p* =  0.368) (Fig. 5).

## Discussion

### Influence of preoperative and postoperative characteristics and middle ear status

The best audiometric results occur when the middle ear is aerated, dry and safe after surgery, so that the ossicular chain − or the material that replaces it − is mobile and stable, as supported by several other studies.^3^, ^4^, ^5^, ^9^, ^10^

Most preoperative characteristics of the patients did not influence the choice of the methods and materials used in the Type 3 Tympanoplasty. The choice was made based on the viability of the stapes structure; if it was mobile and without signs of damage, it was maintained, and the cartilage graft was used. But if the stapes structure was damaged and the footplate was still mobile, the titanium prosthesis was chosen.^3^, ^4^, ^5^, ^8^, ^11^

Some preoperative characteristic − such as patient’s age and cholesteatoma association − intuitively lead us to assume the cartilage graft would be worse than the titanium prosthesis. Kartush^12^ points out that cartilage, as a biological material, is prone to degrade faster. But, by the time of our follow up, there was no sign of cartilage degradation on the audiometric tests.^3^, ^4^, ^5^, ^6^, ^7^, ^8^, ^9^, ^11^, ^12^, ^13^, ^14^, ^15^, ^16^

Also, the postoperative characteristics had no statistically significant difference in the variable group. There was a small difference on the number of patients showing hearing improvement on the titanium group, but it was not statistically significant. The variation probably is a consequence of the reduced number of cases analyzed in this study.^10^, ^12^, ^17^, ^18^

### Audiometric analysis

The analysis of the pure tone audiometry’s results, in different frequencies, showed a statistically significant hearing improvement by decreasing the mean of the air-conduction threshold 6 months after the surgery, but the group factor had no influence on the improvement, once it is comparable for both methods.

The group and frequency factors did not indicate a significant influence in the mean difference, but, analyzing the via factor (air and bone-conduction pathways) the mean of the difference (before and after) showed a greater improvement on air conduction than on bone conduction pathway. This is expected on type 3 tympanoplasty surgeries once it decreases the air bone gap.^6^, ^7^, ^8^, ^9^, ^19^, ^20^

On the air conduction, the sound travels from the air, vibrates the TM graft and all the structures in the middle ear to transmit the acoustic energy to the oval window and consequently to the cochlea. On the other hand, the bone conduction test vibrates the mastoid bone and the skull until the vibration reaches the cochlea, not involving the middle ear structures to conduct the sound. So, the reconstruction of the middle ear structure reestablishes the passage of the sound through the air conduction pathway, correcting the conductive hearing loss and resulting in a significant improvement of the hearing capacity and a reduced air bone gap.^4^, ^5^, ^6^, ^7^, ^19^, ^20^

The air bone gap (A − B gap) is the difference between air conduction and bone conduction threshold at the pure tone average. The air bone gap is positive every time there is a conductive hearing loss, as in our patients with COM. The air bone gap is still tolerated postoperatively once the hearing improvement is significant, but not completely after surgery with both materials. The goal of improvement after tympanoplasty is an air bone gap of ≤ 20 dB, but usually ≤ 30 dB is not a problem for the patient’s normal life.^19^, ^20^, ^21^

Multiple factors may perpetuate the postoperative air bone gap, as Okada et al.^20^ point out: the modifications in the middle ear ossicular structure are massive on both methods, so it can cause some acoustic energy loss along the new material, maintaining a partial hearing loss. The possible TM lateralization, formation of fibrotic tissue around the graft or prosthesis could stiff the system, decreasing mobility and vibration, especially when induced by high frequencies.^20^, ^21^

This is sustained by Nishihara et al.^9^ who tested different amounts of additional mass on the TM, stapes and prosthesis to find out if those additional masses would diminish hearing improvement on some frequencies, mostly high frequencies.^9^

The titanium is less likely to cause a significant addition of weight, as a light material. But the prosthesis has a chance of extrusion, which is small and did not occur in some studies with a short followup. In our study there was no case of prosthesis extrusion.^22^, ^23^, ^24^, ^25^

A complete understanding of the features that influence the hearing improvement and the different postoperative complications would lead us to even better results in the future.

## Conclusion

In this study, the audiometric results of the 26 patients who underwent Type 3 Tympanoplasty Major Columella with Titanium prosthesis or Stapes Columella with cartilage graft showed no statistical significant difference, which indicates that in a short period of time (6 months) the material of choice does not influence the hearing improvement in patients with COM. We also concluded that preoperative and postoperative characteristics did not influence the material chosen. The choice of the method and material was made based on the viability of the stapes structure.

The via factor (air or bone conduction pathway) influences the hearing improvement once the air conduction pathway showed better results, reducing the air bone gap.^6^, ^9^, ^20^

More study on this topic is yet needed. More patients evaluated with a longer followup could clarify some questions that remain. More subjects in the study could show a statistically significant difference between the titanium prosthesis and the cartilage graft groups. Longer follow up could demonstrate better long term hearing outcomes for one or the other technique, even considering the possible causes of bad outcomes – retraction or perforation of the TM graft, prosthesis extrusions – and assist in developing hypothesis of how to avoid those events.^10^, ^14^, ^15^, ^19^^,^^21^

Further studies will be needed to answer all those questions and increase the chance of even better outcomes.

## Conflicts of interest

The authors declare no conflicts of interest.
